# Creative Self-Efficacy, Cognitive Reappraisal, Positive Affect, and Career Satisfaction: A Serial Mediation Model

**DOI:** 10.3390/bs13110890

**Published:** 2023-10-27

**Authors:** Sunyoung Oh, Jungmin Pyo

**Affiliations:** 1 Department of Vocational Studies, Kyonggi University, Seoul 03746, Republic of Korea; 2 Department of Early Childhood Education, Gwangju University, Gwangju 61743, Republic of Korea

**Keywords:** career satisfaction, cognitive reappraisal, creative self-efficacy, emotion regulation, happiness, positive affect, subjective career success

## Abstract

With a substantial body of research supporting the critical role of positive affect in improving work outcomes and enhancing career success, investigating the factors that facilitate emotion regulation strategies for fostering positive affect becomes an important research question. In this context, our study explores the association between strong creative self-efficacy and high cognitive reappraisal—an established and potent emotion regulation strategy known to increase positive affect. We propose a model wherein high levels of creative self-efficacy lead to a tendency for cognitive reappraisal, resulting in high levels of positive affect that ultimately contribute to greater career satisfaction. Our investigation, conducted with a sample of 550 adults in South Korea, examines the indirect relationship between creative self-efficacy and career satisfaction through cognitive reappraisal and, in turn, positive affect. Our findings reveal a positive association between creative self-efficacy and cognitive reappraisal. Moreover, a significant relationship is observed between creative self-efficacy and positive affect through the mediation of cognitive reappraisal. Importantly, the indirect effect of creative self-efficacy on career satisfaction is mediated through cognitive reappraisal and then positive affect. These findings not only expand our insight into the factors contributing to positive affect and career satisfaction but also underscore the valuable role of creative self-efficacy in career satisfaction.

## 1. Introduction

Numerous studies have established a strong link between positive affect and better outcomes in various life domains, including health and work life. For instance, positive affect has been associated with a longer life, improved cardiovascular health, and better cancer survival [1]. Additionally, within workplaces, individuals with higher levels of positive affect not only exhibit enhanced performance but also report greater job satisfaction and career achievement compared to their unhappy counterparts, highlighting the association between positive affect and career success [2,3,4]. Given the benefits of positive affect, cognitive reappraisal has gained increasing attention as an emotion regulation strategy for promoting positive affect, as it has been identified as one of the most effective strategies [5].

Cognitive reappraisal is a strategy for regulating emotions by altering the way a person interprets or understands situations that trigger emotional responses, with the goal of modifying those emotional responses [6]. Numerous studies have demonstrated that experimentally induced cognitive reappraisal has a positive impact on emotional outcomes in response to emotional stimulus [5,7]. Moreover, research has shown that individual differences in cognitive reappraisal tendency are linked to affective functioning and well-being. Individuals with a habitual tendency to use reappraisal more frequently tend to experience more positive affect and less negative affect, greater life satisfaction, and less anxiety and depression than those who use reappraisal less frequently [6,8,9,10].

However, despite the evidence on the association between individual differences in cognitive reappraisal tendency and the experience of positive affect, there is a noticeable gap in empirical research regarding what factors contribute to individual differences in cognitive reappraisal tendency. Our study pivots its attention to the potential relationship between individuals’ creative self-efficacy (i.e., beliefs about their creative ability) and their tendency to engage in cognitive reappraisal. Although cognitive reappraisal is considered an effective emotion regulation strategy, it is often underutilized [11]. Milyavsky et al. [11] suggested that the perceived difficulty of implementing cognitive reappraisal serves as a restraining force that decreases the likelihood of utilizing this strategy. Hence, it is likely that personal characteristics that can function as a personal resource to compensate for this perceived difficulty of cognitive reappraisal may influence an individual’s tendency to use it. Drawing on social cognitive theory [12], which emphasizes the importance of self-efficacy beliefs in motivating actions, and recent research on the relationship between creativity and cognitive reappraisal [13], we propose that having a strong self-efficacy belief in creativity (i.e., high levels of creative self-efficacy) can promote a habitual tendency to use cognitive reappraisal. This, in turn, can contribute to greater career satisfaction through the experience of positive affect. We test this theoretical framework by empirically examining a mediational model in which creative self-efficacy predicts career satisfaction through the sequential mediation of cognitive reappraisal and positive affect. By exploring this relationship, we aim to gain a better understanding of the personal characteristics associated with cognitive reappraisal tendency and expand our insight into the factors contributing to positive affect and career satisfaction.

## 2. Theoretical Background and Hypotheses

Creative self-efficacy refers to an individual’s belief in their ability to generate original ideas and produce innovative outcomes [14]. Tierney and Farmer [14] developed this construct based on Bandura’s [12] definition of self-efficacy perceptions, emphasizing the specific belief individuals have about their creative abilities. Unlike general self-perceptions such as self-esteem or general confidence across domains, creative self-efficacy reflects an individual’s specific task-based judgments about their ability to engage in creative endeavors. Previous research has emphasized that creative self-efficacy is a significant driver of motivation for creative action [15]. Engaging in creative work can be challenging due to anxiety associated with uncertainty and the risk of failure [16]. Creative self-efficacy provides individuals with the inner strength that allows them to remain task-focused. Individuals with strong creative self-efficacy can maintain their persistence in the face of difficulties. In contrast, individuals with low creative self-efficacy may avoid pursuing creative endeavors because they anticipate failure [14]. Consistent with this notion, empirical evidence supports that creative self-efficacy enhances creative performance and innovative behavior [17,18]. For example, Chen and Zhang [19] found that employees’ creative self-efficacy predicted their creative performance, as rated by their supervisor. Ng and Lucianetti [20] conducted a longitudinal study that found a positive relationship between creative self-efficacy and idea generation.

This study extends this line of research to explore the personal characteristics that contribute to career satisfaction. We suggest that individuals’ creative self-efficacy may be associated with their cognitive reappraisal tendency, thus increasing the experience of positive affect, which in turn contributes to career satisfaction. Cognitive reappraisal is a strategy for regulating emotions by altering the way a person perceives or understands a situation that triggers emotional responses, with the goal of modifying those emotional responses [6,21]. Numerous studies have demonstrated that experimentally induced cognitive reappraisal has a positive impact on emotional outcomes in response to emotional stimulus [5,7,21,22]. Moreover, research has shown that individual differences in cognitive reappraisal tendency are linked to affective functioning and well-being [22,23,24]. Individuals with a habitual tendency to use reappraisal more frequently tend to experience more positive affect and less negative affect, greater life satisfaction, and less anxiety and depression than those who use reappraisal less frequently [6,22,25,26,27].

We suggest that creative self-efficacy may be closely associated with the habitual use of cognitive reappraisal in emotion-eliciting situations for several reasons. First, cognitive reappraisal is a complex cognitive process that involves deliberately shifting perspectives or generating alternative perspectives to alter the emotional impact of a situation that elicits emotions [28]. This process necessitates effortful control that requires motivated recruitment of resources. Due to the complexity of creating new reappraisals in emotion-eliciting situations, individuals may perceive cognitive reappraisal as challenging and opt for less demanding regulatory strategies, such as distraction, even though cognitive reappraisal has been shown to have beneficial results in reducing negative emotion [11,29]. According to social cognitive theory, individuals with strong creative self-efficacy beliefs are more likely to persist and approach challenges from different perspectives and generate new ideas, even when faced with difficulties [12,14]. Therefore, it is likely that having a strong creative self-efficacy can compensate for the difficulty of implementing cognitive reappraisal and increase the likelihood of selecting it as a self-generated emotion regulation technique in emotion-eliciting situations. As a result, individuals with high creative self-efficacy are more likely to utilize cognitive reappraisal as an emotion regulation strategy in their daily lives, showing a greater tendency to use cognitive reappraisal. On the other hand, those with low creative self-efficacy are less likely to use this strategy due to the perceived difficulty of cognitive reappraisal.

**Figure 1:**
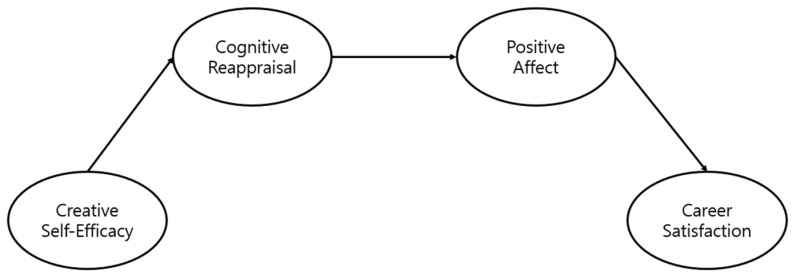
Hypothesized model.

Second, the cognitive processes involved in implementing cognitive reappraisal overlap with those used in conventional creative thinking activities, as both require individuals to flexibly consider an issue from different perspectives and explore alternative interpretations [13]. Furthermore, the effectiveness of reappraisal may rely on the creativity of generated reappraisal [30]. Given the empirical evidence that individuals with high creative self-efficacy tend to demonstrate more creativity [17,31], it is reasonable to suggest that individuals with high creative self-efficacy are more likely to generate creative alternatives for reappraising situations, resulting in more effective emotion regulation. Therefore, individuals with high creative self-efficacy may have a greater tendency to use cognitive reappraisal as a self-generated emotion regulatory strategy in daily life. Accordingly, we predict that individuals’ creative self-efficacy is positively associated with cognitive reappraisal tendency. Additionally, as previous studies have shown that cognitive reappraisal promotes positive affect [21,25], we expect that creative self-efficacy would have a positive relationship with positive affect through cognitive reappraisal (Figure 1). Therefore, we propose the first two hypotheses as follows:

**Hypothesis 1:**  *Individuals’ creative self-efficacy will be positively associated with their cognitive reappraisal.*

**Hypothesis 2:**  *Cognitive reappraisal will mediate the relationship between creative self-efficacy and positive affect.*

Next, we suggest that positive affect may be a mechanism through which creative self-efficacy and cognitive reappraisal contribute to career satisfaction. Career satisfaction or subjective career success refers to an individual’s personal appraisals of their career accomplishments and experiences [32]. Positive affect is widely recognized as a crucial resource for career success, primarily by fostering adaptive work behaviors and promoting the pursuit of career goals [2,4,33]. Previous research provides ample support for this association. For example, Haase et al.’s longitudinal studies [34] indicate that individuals with higher positive affect exhibit greater effort in pursuing their career objectives. Likewise, Ouweneel et al.’s daily diary study [35] showed a positive relationship between positive affect and work engagement across days. In a longitudinal study, Tsai et al. [36] highlighted the motivational effects of positive affect by showing that insurance sales agents with higher levels of positive affect demonstrated more effort in task completion and improved sales performance three weeks later. Furthermore, three empirical reviews [2,4,37] have consistently shown that positive affect has adaptive effects on work-related behaviors and career attainment: Individuals with higher positive affect exhibit greater motivation toward their jobs, more commitment to their employing organizations, better job performance, and more job satisfaction, along with less withdrawal behavior such as absenteeism and turnover. Drawing from these findings and the existing evidence on the influence of cognitive reappraisal on positive affect [6,8,9,10], it is plausible that individuals with a greater tendency for cognitive reappraisal may experience higher positive affect, and, in turn, show greater career satisfaction. We thus hypothesize that individual differences in cognitive reappraisal tendency positively relate to career satisfaction via positive affect. Moreover, we predict that creative self-efficacy will positively correlate to career satisfaction through the pathway of cognitive reappraisal and positive affect (see Figure 1). We, therefore, generate the final two study hypotheses as follows:

**Hypothesis 3:**  *Cognitive reappraisal will have a positive relationship with career satisfaction through positive affect.*

**Hypothesis 4:**  *Creative self-efficacy will have a positive relationship with career satisfaction through cognitive reappraisal and then through positive affect.*

## 3. Materials and Methods

### 3.1. Participants and Procedures

The data presented in this article were collected using the sampling method employed by the second author for his dissertation project. The sampling method used in this study was a student-recruited snowball sampling method. The second author approached students in a large South Korean university to assist in recruiting participants. These students were then encouraged to introduce their acquaintances to this study. This sampling method has been widely used in organizational research to increase generalizability by sampling participants across diverse occupations and demographic backgrounds [38]. Example studies that have employed this method include Grant and Mayer [39], Koopman et al. [40], and Wehrt et al. [41]. In order to enlist undergraduate students to assist in recruiting participants for this study, the second author placed online advertisements on a large university’s bulletin board in Seoul, South Korea. These student volunteers were asked to distribute survey packages and were monetarily compensated for their involvement in this process (for detailed information regarding the sampling procedure, see Supplementary Material File S1).

Through the snowball sampling processes, 585 community adults were recruited. Of the initial 585 participants, we excluded 20 survey responses due to incomplete or invalid responses (for a detailed description of the procedure, see Supplementary Material File S1). In addition, we identified three univariate outliers (±3 SD) and twelve multivariate outliers (Mahalanobis distance with *p* < 0.001) and removed them from the dataset, resulting in a final sample of 550 adults (313 women, 56.9%; 237 men, 43.1%).

The average age of the participants was 41.99 years (SD = 10.69). Regarding education level, 0.2% of participants had completed elementary school, 1.5% had completed middle school, 25.6% had completed high school, 65.1% had completed an undergraduate program, and 7.6% held postgraduate degrees. Gender was coded as 1 for female respondents and 0 for male respondents. Employment status was coded as 0 for those not currently employed and 1 for those employed. For education, the coding was as follows: 1 = elementary school, 2 = middle school, 3 = high school, 4 = bachelor’s degree, 5 = master’s degree or higher.

We conducted a post hoc power analysis using the pwrSET app in Shiny (https://yilinandrewang.shinyapps.io/pwrSEM/, 10 October 2023) by Wang and Rhemtulla [42]. With our sample size and 10,000 simulated samples, the results showed that our sample size was well powered (1.00) to detect the indirect effects of creative self-efficacy on career satisfaction in our serial mediation model.

### 3.2. Measures

To ensure that all participants could understand and respond to the measures, we followed Kwon et al.’s survey translation procedure [43] to translate all English-based measures into Korean.

#### 3.2.1. Creative Self-Efficacy

We assessed participants’ creative self-efficacy using Beghetto’s [44] three-item scale. This scale was developed based on previous work by Tierney and Farmer [14] and Bandura [12]. A sample item is “I am good at coming up with new ideas.” Participants rated each item on a 7-point Likert scale, ranging from 1 (strongly disagree) to 7 (strongly agree). The Cronbach’s alpha was 0.80.

#### 3.2.2. Cognitive Reappraisal

We measured cognitive reappraisal using Gross and John’s [21] scale items rated on a 7-point Likert scale (1 = strongly disagree, 7 = strongly agree). A sample item is, “When I want to feel less negative emotion, I change the way I’m thinking about the situation.” The Cronbach’s alpha was 0.86.

#### 3.2.3. Positive Affect

To assess positive affect, we employed the three-item scale developed by Suh and Koo [45]. Participants indicated how often they had experienced positive affect (e.g., happy) over the past month on a 7-point Likert scale (1 = none of the time, 7 = all of the time). The Cronbach’s alpha was 0.79.

#### 3.2.4. Career Satisfaction

In assessing participants’ career satisfaction, we used a single-item measure of career satisfaction [46,47,48]. Previous research suggests that single-item measures of facet satisfaction (e.g., career satisfaction) comparably perform to multiple-item measures [46,48,49]. Given the complex nature of subjective career success, using a single-item measure may be preferable for measuring overall career satisfaction. This is because single-item measures have more face validity, more aptly capturing the essence of subjective career success than multiple-item measures [49]. Additionally, they are simpler and take less time to complete [49]. Participants rated the item “My career is close to ideal” on a 7-point Likert scale (1 = not at all, 7 = very much).

#### 3.2.5. Control Variables

We measured negative affect using a three-item scale [45]. Based on previous research [34], we also considered controlling for several demographic variables such as age, gender, and education. Multiple regression analyses revealed that negative affect was not a significant predictor of career satisfaction. Age and education were the only significant predictors of career satisfaction, and only age was associated with positive affect and cognitive reappraisal. As a result, we included age and education as additional predictors in all subsequent analyses.

### 3.3. Analytics Strategy

We utilized structural equation modeling in Mplus Version 5.0 [50] to examine the proposed model. Maximum-likelihood estimation was used. Anderson and Gerbing [51] proposed a two-step approach. The first step involves verifying the measurement model to ensure observed variables align with their intended latent constructs, and assessing construct validity. After confirming this model, the second step evaluates the structural model, scrutinizing the relationships between latent constructs based on theoretical or research-driven hypotheses. In line with this methodology, confirmatory factor analyses were executed as the initial phase, succeeded by an evaluation of the hypothesized structural model.

Model fit was evaluated using the comparative fit index (CFI), Tucker-Lewis index (TLI), root mean square error of approximation (RMSEA), and standardized root-mean-square residual (SRMR). To determine an acceptable fit, we followed Hu and Bentler’s suggestion [52] of CFI and TLI values close to 0.95 and RMSEZ and SRMR values close to 0.06 and 0.08, respectively. To test the significance of indirect effects, we used a bias-corrected bootstrap confidence interval (1000 iterations) [53]. We reported unstandardized coefficients and confidence intervals for effect sizes, following the suggestion of Pek and Flora [54].

## 4. Results

Table 1 shows the descriptive statistics and correlations among the study variables. Confirmatory factor analysis was conducted to establish the discriminant validity of the measures of creative self-efficacy, cognitive reappraisal, and positive affect. Each variable was specified as a latent factor represented by its respective measurement items. The three-factor model showed acceptable fit statistics, χ^2^ (51) = 100.24, CFI = 0.98, TLI = 0.98, RMSEA = 0.04, SRMR = 0.03, and all factor loadings were significant. We compared the three-factor measurement model with a single-factor model using a chi-square difference test and the results indicated that the three-factor model had a significantly better fit to the data than the single-factor model, Δχ^2^ (3) = 871.29, *p* < 0.01, thereby supporting the discriminant validity among the three latent constructs.

**Figure 2:**
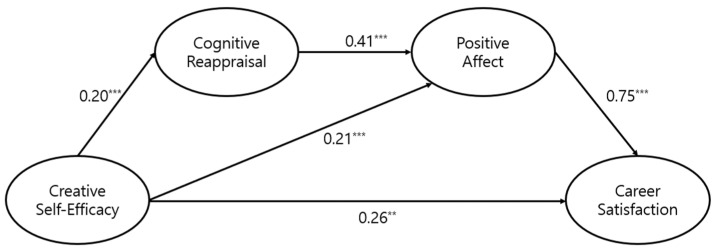
Structural equation modeling results. Notes: Unstandardized coefficients are presented here. Paths of the control variables are omitted for clarity. Age (B = 0.02, SE = 0.01) and education (B = 0.31, SE = 0.08) showed positive relationships with career satisfaction. When relating age to cognitive reappraisal and positive affect, the associations were B = 0.01, SE = 0.002 and B = 0.002, SE = 0.003, respectively). ** *p* < 0.01, *** *p* < 0.001.

To test our proposed model, we first tested a full mediation model (Model 1, as depicted in Figure 1) without any direct paths from creative self-efficacy to positive affect and career satisfaction. The results showed that the proposed model had an acceptable fit to the data, χ^2^ (83) = 236.01, CFI = 0.95, TLI = 0.93, RMSEA = 0.06, SRMR = 0.06. Next, we tested a partial mediation model (Model 2, as depicted in Figure 2) by adding direct paths from creative self-efficacy to positive affect and career satisfaction. This model showed good fit, χ^2^ (81) = 193.62, CFI = 0.96, TLI = 0.95, RMSEA = 0.05, SRMR = 0.04. The direct paths were found to be significant, and their inclusion significantly improved the model fit, Δχ^2^ (2) = 42.39, *p* < 0.01.

We also assessed a partial mediation model with a path from cognitive reappraisal to career satisfaction (Model 3). The direct path was not significant, and this model showed a poorer fit to the data than Model 2, Δχ^2^ (1) = 3.13, ns. Therefore, we adopted the partial mediation model with the direct effects of creative self-efficacy (Model 2, as depicted in Figure 2) as the final model. As depicted in Figure 2, the results of this model supported the hypothesized relationships. Specifically, creative self-efficacy was positively related to cognitive reappraisal (Hypothesis 1), B = 0.20, SE = 0.04, 95% CI [0.12, 0.28], cognitive reappraisal was associated with positive affect, B = 0.41, SE = 0.09, 95% CI [0.25, 0.58], and the relationship between positive affect and career satisfaction was significant, B = 0.75, SE = 0.09, 95% CI [0.58, 0.94].

Furthermore, bias-corrected bootstrapping analyses (1000 bootstrap samples) revealed that the indirect effects from creative self-efficacy to positive affect through cognitive reappraisal were significant (Hypothesis 2), B = 0.08, SE = 0.02, 95% CI [0.05, 0.13], positive affect mediated the relationship between cognitive reappraisal and career satisfaction (Hypothesis 3), B = 0.31, SE = 0.07, 95% CI [0.18, 0.46], and creative self-efficacy had significant indirect effects on career satisfaction through cognitive reappraisal and then through positive affect (Hypothesis 4), B = 0.06, SE = 0.02, 95% CI [0.03, 0.10], total effect = 0.48, SE = 0.07, 95% CI [0.35, 0.66], as shown in Table 2.

Finally, we conducted supplementary analyses. Even though we built our hypotheses upon the theoretical rationale previously explained, we examined alternative models based on Fiedler et al.’s recommendation [55] to test reverse mediation or other causal models for appropriate mediational analyses. To explore the potential reverse relationship between positive affect and career satisfaction, we conducted a non-nested model comparison between our model and an alternative model. The alternative model proposed that creative self-efficacy predicts cognitive reappraisal, which then predicts career satisfaction, and subsequently positive affect (creative self-efficacy → cognitive reappraisal → career satisfaction → positive affect). The fit for this model was characterized by χ^2^ (85) = 310.74, CFI = 0.92, TLI = 0.90, RMSEA = 0.07, SRMR = 0.08. The Bayesian information criterion (BIC) value for this model (BIC = 25,624.23) was larger than that for our final model (BIC = 25,532.34). A lower BIC indicates a better model fit [56], and these results indicate that our final model had a better fit to the data than the reverse causality model. Additionally, we explored the possibility that positive affect might serve as an antecedent of creative self-efficacy by examining another alternative model. In this model, cognitive reappraisal predicted positive affect, which in turn predicted creative self-efficacy, which itself finally predicted career satisfaction (cognitive reappraisal → positive affect → creative self-efficacy → career satisfaction). The fit for this model was characterized by χ^2^ (84) = 2200.91, CFI = 0.95, TLI = 0.94, RMSEA = 0.05, SRMR = 0.06. The BIC value for this model (BIC = 25,540.70) indicated a weaker fit to the data than the final model. Together, these findings support that our theoretically driven model has a better fit for the data than the alternative models.

## 5. Discussion

The purpose of this research was to investigate the relationship between individuals’ creative self-efficacy and their career satisfaction, along with the mediational roles of cognitive reappraisal and positive affect in this relationship. Our findings indicate that (1) individuals with higher creative self-efficacy showed a higher tendency for cognitive reappraisal, and (2) individuals with higher creative self-efficacy reported more positive affect and did so partially through cognitive reappraisal. Furthermore, (3) positive affect is closely associated with career satisfaction, and (4) cognitive reappraisal has a positive relationship with career satisfaction through positive affect. Finally, our results showed that creative self-efficacy has a positive relationship with career satisfaction through cognitive reappraisal and then through positive affect, supporting the following mediation path: creative self-efficacy → cognitive reappraisal → positive affect → career satisfaction.

The present findings have implications for several streams of research. First, our findings contribute to the cognitive appraisal literature by highlighting the significance of creative self-efficacy as an important personal resource that is positively associated with the habitual use of cognitive reappraisal. Milyavsky et al. [11] suggested that although cognitive reappraisal is considered an effective emotion regulation strategy, it is often underutilized due to its perceived difficulty, which serves as a restraining force that decreases the likelihood of utilizing this strategy. Hence, personal characteristics that can function as a personal resource to compensate for this perceived difficulty may influence an individual’s tendency to use cognitive reappraisal. Our results suggest that high creative self-efficacy beliefs can serve as personal resources that increase the motivation to engage in cognitive reappraisal, resulting in its frequent use as a habitual emotion regulation strategy, and consequently contributing to maintaining positive affect in daily life.

Second, our study contributes to the career satisfaction literature by investigating the role of creative self-efficacy and cognitive reappraisal, which has been overlooked by previous research on potential antecedents of career satisfaction. While previous research has identified various personal factors such as cognitive abilities, dispositional personality traits (e.g., Big Five traits), identity, and proactivity as personal resources that promote career satisfaction [32], to our knowledge, none have explored the relationships of creative self-efficacy and cognitive reappraisal to career satisfaction. Thus, these findings broaden our understanding of the personal factors associated with career satisfaction and enrich the existing literature on this topic.

Third, this study contributes to a broader understanding of the role of creative self-efficacy beyond its impact on creative performance. Although there is a growing interest in the adaptive functioning of creative self-efficacy in the workplace, previous research on creative self-efficacy has largely neglected its impact on affect and career satisfaction. Fino and Sun [57] recently proposed that individuals’ creative self-efficacy beliefs can be a valuable resource to help them creatively reframe life stressors and overcome difficult situations, thus promoting mental well-being. Our findings are consistent with their work and suggest that the importance of individuals’ creative self-efficacy is not confined to successful performance in creative and innovative work in organizations, but also encompasses adaptive emotion regulation strategies, such as reappraisal, positive affect, and career satisfaction.

Fourth, our findings hold significant practical implications. These findings indicate that the advantages of employees’ creative self-efficacy extend beyond merely driving innovation; they also may reverberate to areas such as career satisfaction. Hence, organizational strategies focused on enhancing creative self-efficacy are likely to witness multifaceted benefits. Such strategies can lead to not only a boost in creative output but also increase employees’ career satisfaction. An actionable approach in this direction would be to invest in leadership training programs tailored to nurture employees’ creative self-efficacy. Given the pivotal role supervisors play in shaping employees’ self-efficacy, as highlighted by Tierney and Farmer [14], such training can be effective. Moreover, given that Ng and Lucianetti [20] have established the relationship between perceived respect from supervisors and coworkers and a boost in employees’ creative self-efficacy, it is crucial for managers to cultivate a workplace culture rooted in respect, ensuring employees genuinely feel valued.

This study has several limitations. First, we used a cross-sectional, self-report survey, which may have the inherent possibility of common-method bias and limit our ability to draw definitive causal conclusions. Future research could use longitudinal investigations or experimental designs to test the effects of creative self-efficacy on career satisfaction and our proposed mediation model. Second, we used a single-item measure of career satisfaction. Although a single-item global career satisfaction rating is not uncommon in the literature [48], it could be valuable for future research to replicate our hypotheses using a more robust multiple-item career satisfaction scale.

Third, we did not examine the potential moderating effects of work characteristics on the relationship between creative self-efficacy and career satisfaction. Research has shown that work characteristics such as job autonomy and work demands affect job satisfaction, turnover, and well-being [58]. Future researchers could explore whether these factors function as boundary conditions under which creative self-efficacy is more or less likely to predict career satisfaction. Fourth, it is important to acknowledge that our sample of South Korean adults may limit the generalizability of our findings. Although our study involved a diverse group of South Korean community adults from various professional backgrounds, granting us a comprehensive view of career satisfaction across different job roles and sectors in South Korea, one must consider the potential influence of cultural factors. Previous research on country-level relationships between emotion regulation and culture suggests that cultural factors, such as uncertainty avoidance and power distance, may influence cognitive reappraisal tendency [59]. Thus, to validate the applicability of our results to other cultures, future studies should investigate whether the association between creative self-efficacy and cognitive reappraisal varies across different national cultural contexts to test the generalizability of our findings.

## Figures and Tables

**Table 1:** Descriptive statistics and intercorrelations among variables.

Variables	M	SD	1	2	3	4	5	6	7	8
1. Gender	0.57	0.50								
2. Age	41.99	10.69	−0.01							
3. Education	3.79	0.60	−0.13 **	−0.24 **						
4. Employment status	0.78	0.42	−0.36 **	−0.16 **	0.19 **					
5. Creative self-efficacy	4.38	1.06	−0.11 *	0.03	0.13 **	0.12 **				
6. Cognitive reappraisal	4.67	0.89	0.06	0.14 **	0.00	0.01	0.27 **			
7. Positive affect	4.53	0.96	0.06	0.09 *	−0.01	−0.04	0.29 **	0.33 **		
8. Negative affect	3.62	1.11	0.04	−0.15 **	0.00	0.03	−0.06	−0.10 *	−0.45 **	
9. Career satisfaction	4.66	1.33	−0.01	0.15 **	0.14 **	0.07	0.31 **	0.31 **	0.45 **	−0.22 **

Notes: * *p* < 0.05, ** *p* < 0.01.

**Table 2:** Unstandardized path coefficients and indirect effects in structural equation model.

Path	*B*	*SE*	95% CI
Direct effects			
Creative self-efficacy → Cognitive reappraisal	0.20 **	0.04	(0.12, 0.28)
Creative self-efficacy → Positive affect	0.21 **	0.05	(0.12, 0.32)
Creative self-efficacy → Career satisfaction	0.26 **	0.07	(0.11, 0.41)
Cognitive reappraisal → Positive affect	0.41 **	0.09	(0.25, 0.58)
Positive affect → Career satisfaction	0.75 **	0.09	(0.58, 0.94)
Indirect effects			
Creative self-efficacy → Cognitive reappraisal →Positive affect	0.08 *	0.02	(0.05, 0.13)
Cognitive reappraisal → Positive affect → Career satisfaction	0.31 *	0.07	(0.18, 0.46)
Creative self-efficacy → Cognitive reappraisal → Positive affect → Career satisfaction	0.06 *	0.02	(0.03, 0.10)

Notes: * *p* < 0.05, ** *p* < 0.01.

## Data Availability

All the raw data are available from the corresponding author upon request.

## Supplementary Materials

The following supporting information can be downloaded at: https://www.mdpi.com/article/10.3390/bs13110890/s1. File S1: Sampling procedure [60].
