# Supplementary material for: Creative Self-Efficacy, Cognitive Reappraisal, Positive Affect, and Career Satisfaction: A Serial Mediation Model
Source: Behav Sci (Basel). 2023 Oct 27;13(11):890. doi: 10.3390/bs13110890 (PMC10669601; doi:10.3390/bs13110890)
Supplement: Supplementary file 1 [file behavsci-13-00890-s001.zip › behavsci-2616972-supplementary.pdf]

Supplementary Material

Creative Self-Efficacy, Cognitive Reappraisal, Positive Affect and Career Satisfaction: A Serial Mediation Model

Sampling procedure

The data presented in this article were acquired through the sampling method employed by the second author for his dissertation project. The sampling method used in this study was a student-recruited snowball sampling method, which has been widely used in organizational research to increase generalizability by sampling participants across diverse occupations and demographic backgrounds [1-5]. To find undergraduate students to aid in recruiting participants, the second author placed online advertisements on a large university's bulletin board in Seoul, South Korea. These students were asked to distribute survey packages and were compensated monetarily for their involvement in this process. The undergraduate students were asked to recruit both student-groups and community adults, with the specific instructions to enlist two individuals within each of the following five age ranges: 14-19 years, 20-29 years, 30-39 years, 40-49 years, and 50-59 years. This approach was chosen to align with the requirements of the second author's dissertation project, which aimed to encompass a diverse range of samples across different age-groups. Consequently, the sampling methodology led to the enrollment of 585 community adults and 469 students for the dissertation project. However, for the purpose of this study focusing on career satisfaction, only data from adults were utilized. This decision was made as the students primarily identified their occupation as "student", lacking the experiential background necessary for the appraisal of career satisfaction.

Of the initial 585 participants, we excluded 20 survey responses due to incomplete or invalid responses. Among 20 incomplete or invalid responses, eight were incomplete responses, and for the percentage of responses, the range is from 46.7% to 80%. 12 invalid responses were identified by using individual consistency with inter-item standard deviations and person-total correlation, and extreme scores (i.e., ±3) that were highly unlikely to be observed were interpreted as random or careless and therefore invalid responses.6

Regarding the multivariate outliers, we used our final SEM model to compute Mahalanobis distance in Mplus. We tested our final model using a dataset (total N = 585) including the outliers and found that the results were the same as those presented in the main analysis, $\chi2(81) = 134.99$, CFI = 0.97, TLC = 0.96, RMSEA = 0.03, and SRMR = 0.04, indicating that removing the outliers did not change the results.

Raw data

The corresponding author can provide all the raw data upon request.

References

1. Mayer, D.M.; Ong, M.; Sonenshein, S.; Ashford, S.J. The money or the morals? when moral language is more effective for selling social issues. *J. Appl. Psychol.* **2019**, *104*, 1058-1076. https://doi.org/10.1037/apl0000388.

2. Grant, A.M.; Mayer, D.M. Good soldiers and good actors: Prosocial and Impression Management Motives as interactive predictors of affiliative citizenship behaviors. *J. Appl. Psychol.* **2009**, *94*, 900-912. https://doi.org/10.1037/a0013770.
3. Koopman, J.; Scott, B.A.; Matta, F.K.; Conlon, D.E.; Dennerlein, T. Ethical leadership as a substitute for Justice Enactment: An information-processing perspective. *J. Appl. Psychol.* **2019**, *104*, 1103-1116. https://doi.org/10.1037/apl0000403.
4. Wehrt, W.; Casper, A; Sonnentag, S. Beyond depletion: Daily self-control motivation as an explanation of self-control failure at work. *J. Organ. Behav.* **2020**, *41*, 931-947. https://doi.org/10.1002/job.2484.
5. Wheeler, A. R.; Shanine, K. K.; Leon, M. R.; Whitman, M. V. Student-recruited samples in organizational research: A review, analysis, and guidelines for future research. *J. Occup. Organ. Psychol.* **2014**, 87, 1-26. https://doi.org/10.1111/joop.12042.
